# Maltreated and non-maltreated children’s truthful and dishonest reports: Linguistic and syntactic differences

**DOI:** 10.3389/fpsyg.2022.1025419

**Published:** 2022-12-14

**Authors:** Victoria W. Dykstra, Thomas D. Lyon, Angela D. Evans

**Affiliations:** ^1^Psychology Department, Brock University, St. Catharines, ON, Canada; ^2^Gould School of Law, University of Southern California, Los Angeles, CA, United States

**Keywords:** lie detection, maltreatment, verbal cues, linguistic, syntax, deception

## Abstract

**Introduction:**

Adults are typically poor judges of the veracity of statements, requiring the need for alternative methods for detecting lies. One alternative method to human lie-detectors is using computer-based linguistic analysis which may present a more reliable method for detecting dishonesty. Moreover, while previous research has examined linguistic differences between typically developing children’s and adults’ truthful and dishonest reports, no study to date has examined whether maltreated children exhibit different linguistic cues to dishonesty. Thus, the current study examined maltreated and nonmaltreated children’s linguistic and syntactic cues to children’s truthful and dishonest reports.

**Methods:**

Nine- to 12-year-olds, half of whom were maltreated, played a computer game with a confederate: half of the children experienced a transgression (i.e., playing a forbidden game and crashing the computer) and were coached to conceal it, and half of the children experienced no transgression (i.e., simply played a computer game). All children were then interviewed about the event. The current study utilized automated linguistic and syntactic analysis software to compare children’s truthful reports (no transgression occurred) with dishonest reports.

**Results and Discussion:**

Results indicated that maltreated and non-maltreated children did not differ in their indicators of dishonesty. Dishonest reporters used more first-person plural pronouns and cognitive mechanism terms and had less syntactically complex reports compared to truthful reporters. Finally, first-personal plural pronouns, cognitive mechanism terms, and syntactic complexity accurately classified (74.2%) the veracity of children’s reports. The current findings present a new indicator of dishonesty (syntactic complexity) and suggest that indicators from typically developing populations may apply to maltreated children when coaching occurred.

## Introduction

The ability to identify children who are dishonest about or reluctant to disclose negative experiences has important implications in forensic contexts. For example, failing to identify children who conceal maltreatment can lead to a child being left in a harmful environment. This can lead to further abuse, resulting in negative developmental outcomes including internalizing and externalizing problems (Vilariño et al., 2022). Establishing markers of dishonesty in cases where a child may be concealing some details while falsifying others may assist in providing a tool for professionals to identify cases that may require further investigation. One potential method for identifying dishonesty is assessing verbal differences in honest and dishonest reports. In fact, previous research suggests that verbal cues may be more reliable and accurate than non-verbal cues when attempting to detect children’s dishonest reports, given that truth-and lie-tellers do not differ on many non-verbal cues to deception (e.g., eye movement, body language; Talwar and Lee, 2002). While progress has been made in identifying verbal markers of deception with typically developing children, no study to date has examined whether these markers are also relevant for maltreated children. Given that maltreated children often experience delays in language development (Rogosch et al., 1995; Geeraert et al., 2004) they may exhibit different verbal cues than their typically developing peers. Thus, the aim of the current study was to examine linguistic and syntactic cues to dishonesty (when children are coached to falsify details to conceal a transgression) in maltreated and non-maltreated children’s reports of an adult interaction.

Current research examining linguistic cues to dishonesty with children has primarily utilized paradigms in which children provide reports of a true event as well as a false event after being coached by a parent or researcher. These reports are then compared for linguistic cues that can be used to differentiate the veracity of the statements (Bruck et al., 2002; Evans et al., 2012; Brunet et al., 2013; Williams et al., 2014; Talwar et al., 2018). For example, Evans et al. (2012) and Saykaly et al. (2013) had children play a game with an experimenter where stickers were placed on the child’s body (e.g., their arm). The children were also coached by a parent to falsely report playing an additional game they had *not* played. As such, these studies compared children’s reports of a true experience to fully fabricated reports. However, when being dishonest children may not always completely falsify an event; they may falsify some details to conceal true aspects of the event. There may be different cues to dishonesty when children are coached to conceal only a portion of a true event by providing false information instead, such as a transgression that occurs within the event. Such reports are distinct in several important ways. First, children’s dishonesty is motivated by a desire to avoid a negative consequence of a transgression, rather than providing a story about a neutral event without consequence. Second, children are only told to be dishonest regarding a *portion* of the event; they can reveal some details but must monitor their reports to withhold the details that must be concealed. While they are monitoring what to conceal, they must also provide the coached falsified details. The increased complexity of this task as well as the motivation behind it may lead to different linguistic or syntactic patterns. Importantly, being able to detect instances when children falsify some details to conceal a transgression would be particularly useful for interviewing children about serious events, such as maltreatment.

### Linguistic cues to children’s dishonesty

According to the Activation-Decision-Construction-Action Theory (ADCAT; Walczyk and Fargerson, 2019) telling a lie is a cognitively demanding task, making it difficult to conceal potential markers to deception. First, when a question is asked, working memory is activated to hold the truth in the mind. If the decision to lie is made, the lie-teller must inhibit the truth and construct a plausible alternative response. During the construction of the lie, theory of mind is required to understand the recipient’s knowledge or belief to construct a believable lie. Finally, the action stage involves providing the constructed lie to the recipient while monitoring any verbal or non-verbal cues that might reveal the lie (Walczyk et al., 2003, 2009, 2014). Given the many cognitive abilities at work while lying, children may find it difficult to monitor verbal cues that may reveal their lie. Below we review the relevant literature on linguistic differences between children’s honest and deceptive reports.

One goal when lying is to distance the self from the lie, resulting in the observed reduction of first-person pronouns in adults’ dishonest statements (Hauch et al., 2015). However, studies examining linguistic cues to children’s dishonesty have found children’s lies tend to include *more* self-references (first-person pronouns) compared to truthful statements (Brunet et al., 2013; Williams et al., 2014; Talwar et al., 2018). Importantly, previous research often examines total self-references as a combination of singular (e.g., I, me) and plural (e.g., we, our) pronouns (Brunet et al., 2013; Talwar et al., 2018). Williams et al. (2014) parsed apart these findings by examining singular and plural pronouns separately and found that children who were coached to fabricate stories about events (e.g., sports, parties) used more first-person *plural* pronouns than those who truthfully reported; they did not find differences in the use of singular pronouns. One possible explanation for this increase in first-person plural pronouns in particular when being dishonest may be that children are attempting to disperse blame or responsibility (Talwar et al., 2004; Evans et al., 2021) for the dishonest statement or actions. This may be particularly relevant when children are coached, or a transgression has occurred.

Another theoretical difference between reports of true and fabricated events is the processes used to provide the report. The Reality Monitoring approach to deception detection stipulates that there are different processes that govern reports of truly experienced events compared to fabricated ones. Specifically, truly experienced events are formed based on external experiences and information, while untrue events are internally formulated based on thoughts or cognition. Given this, reports of these events should contain information that demonstrates these processes (Johnson and Raye, 1981). Specifically, recalling true experiences should theoretically rely on external memory attributes, such as sensory and affective processes, because the description is based on real memories of places, events, and emotions (Vrij et al., 2004; Strömwall and Granhag, 2005). In contrast, reporting an untrue event may contain more internal memory attributes, such as cognitive information; thus, the language used to fabricate information may contain more cognitive and fewer sensory and affective words. In adults, using the reality monitoring criteria has been found to effectively differentiate between true and fabricated reports (Vrij et al., 2000; Granhag et al., 2001; Oberlader et al., 2016); however, some of the individual scales, such as affective information, have not been found to uniquely differentiate true and false reports (DePaulo et al., 2003; Masip et al., 2005; Gancedo et al., 2021).

Despite findings that affective and cognitive information may not uniquely identify dishonest reports in adults, previous research examining children’s language suggests that the presence of cognitive or affective words may differ in true and false reports. Children tend to use more cognitive terms in dishonest than truthful statements (Vrij et al., 2004; Williams et al., 2014; Talwar et al., 2018), which supports the notion that lying relies more heavily on internal memory attributes such as cognitive processes. Additionally, research with children supports the idea that reports of true memories rely on external memory attributes to describe true experiences; children tend to use more affect (emotion) words when describing true events compared to false ones (Masip et al., 2005; Williams et al., 2014). In fact, Williams et al. (2014) found that 4-to 7-year-old children who provided false reports about typically occurring events (e.g., sports, birthday parties) used fewer positive and negative emotion words compared to children who told the truth. However, contrary evidence suggests that children may use emotion words when being dishonest, but may lack the ability to describe emotions that are relevant to the event they are lying about; for example, children’s false reports about a serious injury contained more positive emotion words than truthful reports (e.g., breaking a bone; Warren et al., 2018).

The final three word types of interest (tentative, exclusion, and negation terms) have either not been found to differ or have not yet been examined in studies exploring linguistic differences in children’s truthful and dishonest reports. While adult lie-tellers have been shown to use *fewer* tentative (Hauch et al., 2015) and exclusion terms (Newman et al., 2003; Bond and Lee, 2005; Schelleman-Offermans and Merckelbach, 2010; Hauch et al., 2015), studies examining exclusion terms in children’s reports have failed to find significant differences (Brunet et al., 2013; Williams et al., 2014). Tentative terms may be avoided by lie-tellers because they suggest that the lie-teller is not confident about their narrative. Similarly, exclusion words (e.g., but, except, and without) may suggest that the lie-teller is presenting conflicting information and, thus, are also avoided. Adults have been found to use more negation terms when lying compared to telling the truth (Ali and Levine, 2008; Hancock et al., 2008; Hauch et al., 2015). This may also be the case among children as they may use negation terms to ensure the interviewer that nothing bad happened, particularly when being dishonest to conceal a transgression (e.g., “*Nothing* bad happened” or “He did that *without* me”). However, negation terms have not yet been examined in children’s reports.

### Syntactic cues to dishonesty

In addition to the linguistic features of a report, the number of words and syntactic complexity (range and sophistication of the structures that make up sentences; Van Valin, 2001; Ortega, 2003) may also help identify children’s dishonesty or reluctance to disclose. There is some evidence that both adult and child lie-tellers tend to keep their story simple and ambiguous to avoid leaking incriminating details (Vrij et al., 2010; Gongola et al., 2021). If they provide less information, it is easier to maintain the lie across questions or time. However, previous research has found inconsistent support for whether children’s reports differ in length (word count); some studies find that lie-tellers’ reports are shorter than truth-tellers’ reports (Brunet et al., 2013), while others find no difference (Evans et al., 2012; Saykaly et al., 2013). Importantly, Brunet et al. (2013) asked children to provide truthful or fabricated reports of a stressful event (i.e., true or fabricated reports of being bullied), without being coached, and found that truth-tellers’ reports were longer than lie-tellers’. In contrast, the studies that found no differences between truthful and fabricated reports included parental coaching. Thus, coaching may enable children to provide enough information to match the length of their report to truth-tellers, though this pattern has not yet been examined in the context of lying to conceal a transgression. Further investigation is required to more completely understand the influence of coaching on the length of children’s dishonest reports specifically when they have been coached to falsify and conceal details to cover a transgression.

Another cue that may be influenced by the cognitive load of deception is the syntactic complexity of sentence structures. The syntactic sentence structure refers to the rules that govern the ways in which words are arranged within a sentence in a given language (Van Valin, 2001; Ortega, 2003). Previous research has yet to examine (in adults or children) whether the complexity of sentence structure within a report is an indicator of deception. As previously mentioned, lie-telling is a cognitively demanding task for young children (Walczyk et al., 2003, 2009); this complexity may require cognitive resources that limit lie-tellers’ abilities to produce more complex sentence structure. This may be especially true for children as they require greater cognitive resources to employ the cognitive functions involved in lie-telling, leaving less resources available for syntactic complexity. Truth-tellers, by comparison, only need to focus on conveying the relevant information. Because they do not need to focus on the additional tasks of inhibiting the truth and fabricating plausible details, truth-tellers may use more complex sentence structure in their reports. Furthermore, like the overall length of the report, it is possible that coaching may reduce some of the cognitive load that children experience when dishonestly reporting on an event, and therefore lie-tellers may be able to match their syntax to that of truthful reports when coached.

### Maltreatment

The limited research exploring linguistic cues to dishonesty has solely focused on typically developing populations. However, the linguistic cues used to identify dishonesty with typically developing children may not apply to other populations, such as maltreated children, who tend to exhibit delays in language development (Rogosch et al., 1995; Geeraert et al., 2004). Compared to their non-maltreated peers, maltreated children learn fewer words (Coster et al., 1989; Beeghly and Cicchetti, 1994) and exhibit poorer performance on measures of expressive language (for meta-analysis see Sylvestre et al., 2016). Furthermore, there is evidence beginning in early childhood that maltreated children produce less complex utterances compared to non-maltreated children (Coster et al., 1989; Beeghly and Cicchetti, 1994; Eigsti and Cicchetti, 2004). Thus, even with coaching, maltreated children may not exhibit the same linguistic patterns between truthful and dishonest reports as their peers due to delayed language development.

### Honesty promotion

While identifying dishonesty is one method for ensuring reluctant children are identified, another method is to support children in truthfully reporting their experiences. To date, there are several honesty promotion techniques that have been shown to be useful to encourage children to provide truthful reports of transgressions including the putative confession (Lyon et al., 2014; Rush et al., 2017; Cleveland et al., 2018; Quas et al., 2018; Evans and Lyon, 2019). The putative confession involves the interviewer telling the child that their co-transgressor has already told the interviewer everything that happened and wants the child to tell the truth. Across numerous studies, this technique has been found to be effective in increasing honesty with children 4-to 10-years of age (Lyon et al., 2014; Rush et al., 2017; Stolzenberg et al., 2017; Quas et al., 2018; McWilliams et al., 2021). While this method encourages honesty, it may also influence the language children use within their reports. Specifically, children’s cognitive load is increased by this statement because the child not only needs to provide a report, but also has to think about what their co-transgressor may have reported. This increased monitoring may be more cognitively taxing and influence the linguistic and syntactic makeup of children’s reports.

### The current study

The current study examined linguistic and syntactic cues to 9- to 12-year-old maltreated and non-maltreated children’s dishonest reports to conceal a transgression, as well as the potential influence of the putative confession on those reports. Specifically, we examined whether truthful and dishonest reporters differed in linguistic and syntactic cues. Additionally, we examined whether the linguistic and syntactic cues differed based on honesty promotion technique (the putative confession vs. no honesty promotion) technique, age, and maltreatment status. The present study used a forensically relevant paradigm where children were involved in a co-transgression with an adult and were coached to conceal it.

To identify potential markers of dishonesty, truthful reporters (*n* = 164) were compared to dishonest reporters (who lied about the transgression; *n* = 84). Linguist Inquiry Word Count (LIWC; Pennebaker et al., 2001) software was used to analyze the frequency of singular (e.g., I, me) and plural (e.g., we, our) first-person pronouns, cognitive mechanism terms (e.g., cause, know, and ought), affect terms (e.g., happy, worry, and sad), tentative terms (e.g., maybe, perhaps, and guess), exclusive terms (e.g., but, without, and exclude), and negations (e.g., no, not, and never), as well as the overall word count. Connexor Machinese Syntax Software (Samuelsson and Voutilainen, 1997) was used to analyze the syntactic structure of each sentence in children’s reports.

### Hypotheses

#### Honesty

The first set of predictions focused on linguistic differences between truthful and dishonest reporters. First, it was predicted that compared to truthful reporters, dishonest reporters would use more first-person pronouns (examined singular and plural; Brunet et al., 2013; Williams et al., 2014; Talwar et al., 2018) and cognitive mechanism terms (H1; Williams et al., 2014; Talwar et al., 2018). Second, it was predicted that compared to truthful reporters, dishonest reporters would use fewer affect terms (negative and positive emotion words; Masip et al., 2005; Williams et al., 2014), tentative terms (Hauch et al., 2015), and exclusive terms (H2; Newman et al., 2003; Bond and Lee, 2005; Schelleman-Offermans and Merckelbach, 2010; Hauch et al., 2015). Finally, it was predicted that compared to truthful reporters, dishonest reporters would use more negations (H3; Ali and Levine, 2008; Hancock et al., 2008; Hauch et al., 2015).

The second set of predictions examined differences in the length and complexity of truthful and dishonest reporters. First, it was predicted that dishonest reporters would provide significantly shorter reports than truthful reporters (i.e., higher word count, H4; Vrij, 2005; Brunet et al., 2013). Second, while previous research has not yet examined syntactic complexity as an indicator of dishonesty, we expected honest reports to be more complex, while dishonest reporters’ reports would be less complex due to the greater cognitive load associated with lie-telling (H5; Walczyk and Fargerson, 2019).

Importantly, dishonest reporters received coaching regarding details about the game they were supposed to play. Research has shown that linguistic differences tend to disappear when children receive coaching (e.g., first-person pronouns, cognitive mechanism terms; Talwar et al., 2018). However, this has not yet been examined in maltreated samples. We examined the possibility that the linguistic differences between coached dishonest reporters and truthful reporters described above may only emerge in the maltreated sample (H6). The language delays experienced by maltreated children may make it more difficult for coaching to eliminate or minimize linguistic differences between true and false reports.

#### Developmental differences

We also examined developmental differences among the indications of interest, beginning with age differences. There is limited evidence that young children use more emotion words in their reports (Williams et al., 2014), thus we predicted we might also find that younger children use more emotion words than older children (H7). Furthermore, we expected that older children’s reports would be longer and more syntactically complex than younger children’s reports (H8). No other age differences were predicted for linguistic or syntactic differences as there has been no support for such predictions in previous findings in our participants’ age range.

Given that maltreatment is related to delayed language development (Rogosch et al., 1995; Geeraert et al., 2004; Sylvestre et al., 2016), we expected maltreated children would provide shorter reports and use significantly less complex syntactic structure compared to non-maltreated children (H9).

#### Honesty promotion

Tentative, exclusive, and negation terms in particular may be influenced by the putative confession. Children who believe their co-transgressor told the interviewer about the transgression may be uncertain about what details to provide. Thus, they may be more likely to use tentative and exclusive terms in their report. Additionally, they may be even more adamant that they are not to blame for the transgression and may be more likely to use more negation terms to avoid blame (Honesty Promotion predictions = H10).

## Materials and methods

### Participants

A total of 321 9- to 12-year-olds (*M* = 10.50, *SD* = 1.12, 153 males) participated in the original study (Evans and Lyon, 2019). Given that the current study was interested in differences between truthful reporters (no transgression) and dishonest reports (children who lied about the transgression), the children who were in the Break condition and truthfully disclosed the transgression were excluded. Thus, a total of 248 children were included in the current study.

Half of the children were maltreated (*N* = 124, 64 9–10-year-olds, *M* = 7.45, *SD* = 0.50, 33 males; 60 11–12-year-olds, *M* = 11.47, *SD* = 0.50, 31 males). Maltreated children were recruited from the Los Angeles County dependency court. Given that children were removed from parental custody due to substantiated cases of abuse or neglect, the Presiding Judge of Juvenile Court and the Los Angeles County Children’s Law Center granted consent. Maltreated children were ineligible if they were awaiting an adjudication or contested disposition hearing on the date of testing (because they might be asked to testify) or if interpreter services were provided to their family and they were unable to communicate with the researchers in English. The sample was 56.5% Latino, 27.4% African American, 8.8% Caucasian, and 7.3% other. The non-maltreated sample was recruited from schools in mainly low-income ethnic minority neighborhoods (*N* = 124, 67 9–10-year-olds, *M* = 9.49, *SD* = 0.50, 33 males; 57 10–11-year-olds, *M* = 11.42, *SD* = 0.50, 25 males). Ethnic background was comparable to the maltreated sample: 58.9% Latino, 37.1% African American, 1.6% Caucasian, and 4% other. Non-maltreated children’s parents provided written consent and all children provided verbal assent prior to participating. All study procedures were approved by the University of Southern California’s Institutional Review Board.

### Procedure

#### Transgression paradigm

Children began by completing several tasks unrelated to the current study with a female interviewer for approximately 10 min. Following the completion of these tasks, a male confederate entered the room to complete a video game activity. The female interviewer introduced the child to the male confederate and explained that when she returned, she would ask the child some questions about the video game they played while she was gone. She then left the room. The confederate opened a laptop to play one of two games: the Ball game or the Jewel game (the game played was counterbalanced between participants).

All children were randomly assigned to either the Break or No-Break Control condition. The confederate told children in the Break condition that he had played the game they were supposed to play too many times and wanted to play a different game instead. During the game, the confederate noted eight target details for the child to remember (e.g., “Check out the *birds*”). After 2 min, the confederate told the child to click a square that resulted in the computer crashing (a blue error screen appeared), following which the confederate explained they were not supposed to play the game because the computer crashes and the data on the computer was lost. He then explained to the child that the female interviewer was his boss and would be coming back to ask about the game they played. He asked the child to keep secret the fact that they had played the forbidden game and coached the child on details to provide during the interview. Specifically, he told them not to mention 4 details about the game they had played (e.g., “Do not say that there were *birds*”) and provided 4 details they should mention about the game they were supposed to have played (e.g., “Say you saw *blocks falling*”). The confederate then closed the computer and left the room.

In the No-Break Control condition, the child and confederate played a video game that did not cause the computer to crash. The confederate pointed out the same 8 target details for the game they played. After they finished the game, he said that the female interviewer would be returning to ask the child about the game. He then closed the computer and left the room.

#### Interview

Children’s interviews were designed to be similar to best practice forensic interviews, with the use of rapport building and initial use of broad open-ended requests for recall, similar to the National Institute of Child Health and Human Development (NICHD) Structured Protocol, an internationally used evidence-based protocol for forensic interviews with children (Lamb et al., 2007).

##### Rapport phase

The female interviewer from the beginning of the session returned to the room. She began the interview with a 2-min rapport-building phase by asking the child to talk about the last time he or she felt really good or bad at school.

##### Recall

The recall phase began with an instruction based on one of two honesty conditions: Putative Confession or Control. In the control condition, the interview began with the following instruction: “Now that I know you a little better, [child’s name], tell me everything that happened while I was out of the room from the very beginning to the very end.” In the Putative Confession condition, children were told, “Now that I know you a little better, [child’s name], *let me tell you something. The man, [confederate’s name], who came in here, told me everything that happened and he said he wants you to tell the truth*. Tell me everything that happened while I was out of the room from the very beginning to the very end.” Interviewers used facilitators (e.g., “*uh-huh*”) and additional prompts (e.g., “*What happened next?*”) to encourage the child to continue until they completed their initial narrative. Children were then asked what the first thing that happened was followed by a series of *what happened next* prompts until the child exhausted their narrative (*M_prompts_* = 2.75, SD = 2.35). The interviewer then used two follow-up open-ended prompts [e.g., “You said (action/verb). Tell me more about (action/verb).”]. Finally, children were asked to tell the interviewer everything they heard and everything they saw while the interviewer was gone (2 separate questions).

Two groups of children were included in the study based on their condition and their disclosure during the interview phase. In the Break condition, only children who concealed the transgression, dishonest reporters, were included (children who disclosed were not). The second group included children who were in the No-Break Control condition. These groups were chosen to compare because children who truthfully reported the event where no transgression occurred (No-Break Control) and children who experienced the transgression but concealed it (dishonest reporters in Break Condition) provided similar reports of the event. Specifically, both describe an event during which they played a computer game, but only one group is honestly reporting that event. Thus, the truthful reporters (No-Break Control) and dishonest reporters (Break) were compared in the current study.

### Software analysis

Each child’s interview was transcribed verbatim to be analyzed by two software programs.

#### Linguistic inquiry word count

LIWC software is designed to analyze words within a transcript and code them into word categories (Pennebaker et al., 2001). Each word is compared to the words within the program’s internal library and subsequently placed into the relevant word categories. The output provides a frequency with which each word category was used within the report. For the present study, we focused on 7 of these word categories [first-person singular (e.g., I, me) and plural pronouns (e.g., we, our), cognitive mechanism terms (e.g., cause, know, and), affect terms (e.g., happy, worry, and sad), tentative terms (e.g., maybe, perhaps, and guess), exclusive terms (e.g., but, without, and exclude), and negations (e.g., no, not, and never)]. Additionally, LIWC provides a count of the total words within the transcript. The reliability of the word categories used in the current study range from α = 0.43–0.67 (note: evaluating behavior, such as language, is distinct from evaluating psychological measurement; acceptable internal consistency for word types is lower given that repetition typical of psychological measures is not present in verbal behaviors; Boyd et al., 2022).

#### Connexor machinese syntax software

Connexor software was used to analyze the syntactic complexity of children’s reports. It also produces a syntax tree to represent the complexity of the sentence structure itself, which is what is used in the current study to determine the syntactic complexity of children’s reports. The software output provides the number of layers in each sentence within the transcript, which represent the number of noun and verb phrases in each sentence. Connexor’s syntactic accuracy is 93.5% (Samuelsson and Voutilainen, 1997).

Each transcript was analyzed using the Connexor program to obtain the number of layers per sentence for each child’s report. We then calculated the mean number of layers used per sentence across the report for each child. This mean was used in the analyses to represent syntactic complexity, such that a higher score indicated that the child’s sentences were more complex.

## Results

All analyses were conducted using SPSS (v28). First, to ensure univariate normality and remove extreme outliers, we performed a square-root transformation on all dependent variables. We assessed multivariate normality by calculating Mahalanobis distance for each participant’s scores and comparing the highest value to the critical chi square table (Pallant, 2007). With nine dependent variables, values above 27.88 are considered outliers. Two participants in our dataset were above this value (max value = 28.70); however, given that these participants were above the critical value by less than 1, we decided to retain these data points as has been done in previous research (e.g., Hashemian et al., 2012).

Differences between groups on word types and syntactic complexity were assessed using a 4 (Age: 9, 10, 11, 12) by 2 (Honesty: Truthful Reporters vs. Dishonest Reporters) by 2 (Maltreatment Status: Maltreated vs. Non-Maltreated) by 2 (Honesty Promotion: Putative Confession vs. Control) MANOVA. The outcomes of interest were square-root transformed first-person pronouns, cognitive mechanism, affect, tentative, exclusive, and negation terms, as well as word count and complexity (average number of layers in children’s sentences). The MANOVA revealed significant main effects of Age, *F*(27, 630) = 1.89, *p* = 0.005, η_p_^2^ = 0.075, Maltreatment Status, *F*(9, 208) = 2.07, *p* = 0.034, η_p_^2^ = 0.082, Honesty, *F*(9, 208) = 5.42, *p* < 0.001, η_p_^2^ = 0.19, and Honesty Promotion, *F*(9, 208) = 2.98, *p* = 0.002, η_p_^2^ = 0.114, as well as an Age by Honesty by Maltreatment Status by Honesty Promotion interaction, *F*(27, 630) = 1.52, *p =* 0.046, η_p_^2^ = 0.061. Below we outline each significant main effect and interaction in turn.

### Main effect of honesty

Supporting H1 and H5, there was a significant main effect of honesty on first-person plural pronouns, *F*(1, 216) = 4.58, *p* = 0.033, η_p_^2^ = 0.021, cognitive mechanism terms, *F*(1, 216) = 5.23, *p* = 0.023, η_p_^2^ = 0.024, and complexity, *F*(1, 216) = 39.87, *p* = < 0.001, η_p_^2^ = 0.16. Dishonest reporters used *more* first-person plural pronouns than truthful reporters (dishonest reporters: *M* = 1.46, *SD* = 0.56; truthful reporters: *M* = 1.31, *SD* = 0.51; e.g., dishonesty reporter: “*we* just played and he just told me um, helped me when I needed help”). Dishonest reporters used *more* cognitive mechanism terms than truthful reporters (dishonest reporters: *M* = 4.24, SD = 0.44; truthful reporters: *M* = 4.08, SD = 0.44; e.g., dishonest reporter: “I only *know* the beginning and then he put away the laptop”). Additionally, dishonest reporters’ statements were *less* complex than truthful reporters’ (dishonest reporters: *M* = 1.79, SD = 0.13; truthful reporters: *M* = 1.94, SD = 0.14), supporting the prediction that dishonest reporters would use less complex syntax in their reports (H4). The main effect of honesty on first-person plural pronouns was qualified by the significant interaction (discussed below). Hypotheses 2 and 3 regarding differences on affect, tentative, and negation terms, as well as H4 regarding word count, were not supported. Additionally, contrary to H6, the above effects were not impacted by maltreatment status.

### Developmental differences

#### Main effect of age

There was a significant main effect of age on complexity, *F*(3, 216) = 4.53, *p* = 0.004, η_p_^2^ = 0.059, first-person plural pronouns, *F*(3, 216) = 3.35, *p* = 0.020, η_p_^2^ = 0.044, and tentative terms, *F*(3, 216) = 3.24, *p* = 0.023, η_p_^2^ = 0.043. Post-hoc tests using Bonferroni correction were used to examine specific age differences. Partially supporting H8, 12 year-olds’ reports (*M* = 1.96, *SD* = 0.15) were significantly more complex than 9 year-olds’ reports (*M* = 1.86, *SD* = 0.16), *p* = 0.002, and 10 year-olds’ reports (*M* = 1.85, *SD* = 0.15), *p* < 0.001. When solely examining first-person plural pronouns and tentative terms, no significant differences emerged between ages. However, the main effect of age on first-person plural terms was qualified by the significant interaction (discussed below). Contrary to H8, no age differences in the use of affect terms emerged.

#### Main effect of maltreatment status

Supporting H9, there was a significant main effect of maltreatment on complexity, *F*(1, 232) = 6.05, *p* = 0.015, η_p_^2^ = 0.027, and word count, *F*(1, 216) = 3.92, *p* = 0.049, η_p_^2^ = 0.018. Non-maltreated children had *more* complex statements (non-maltreated: *M* = 1.91, SD = 0.15; maltreated: *M* = 1.86, SD = 0.16), and used *more* words (non-maltreated: *M* = 18.38, SD = 4.29; maltreated: *M* = 17.66, SD = 4.56) than maltreated children. Additionally, there was a main effect of maltreatment on the use of affect terms, *F*(1, 216) = 5.63, *p* = 0.019, η_p_^2^ = 0.025, and negation terms, *F*(1, 216) = 3.97, *p* = 0.048, η_p_^2^ = 0.018. Specifically, maltreated children used *more* affect terms (maltreated: *M* = 0.83, SD = 0.22; non-maltreated: *M* = 0.78, SD = 0.21) and negation terms (maltreated: *M* = 0.89, SD = 0.55; non-maltreated: *M* = 0.76, SD = 0.47) than non-maltreated children.

### Main effect of honesty promotion

There was a significant effect of honesty promotion on first-person singular terms, *F*(1, 216) = 6.18, *p* = 0.014, η_p_^2^ = 0.028, affect terms, *F*(1, 216) = 7.76, *p* = 0.006, η_p_^2^ = 0.035, complexity, *F*(1, 216) = 4.34, *p* = 0.038, η_p_^2^ = 0.020, and word count, *F*(1, 216) = 6.36, *p* = 0.012, η_p_^2^ = 0.029. Children in the Putative Confession condition used *more* first-person singular pronouns (putative confession: 2.12, SD = 0.54; control: M = 1.99, SD = 0.56), and affect terms (putative confession: *M* = 0.83, SD = 0.22; control: *M* = 0.78, SD = 0.21) than children in the control condition. Children’s reports in the Putative Confession condition were *more* complex (putative confession: *M* = 1.91, *SD* = 0.16; control: *M* = 1.86, *SD* = 0.15), and contained *fewer* words (putative confession: *M* = 17.21, SD = 4.35; control: *M* = 18.68, SD = 4.41). Beyond these differences, the predicted differences between Honesty Promotion conditions were not supported (H10).

### Interaction

The main effect of honesty and age on the use of first-person plural pronouns were qualified by a significant 4-way interaction (Honesty x Age x Maltreatment Status x Honesty Promotion), *F*(27, 630) = 1.52, *p =* 0.046, η_p_^2^ = 0.061. To examine the effect of the interaction on first-person plural pronouns, follow-up univariate ANOVA were conducted. First, the effect of Honesty, Maltreatment, and Age were examined separately for each Honesty Promotion condition. In the control condition, there was a significant main effect of Honesty, *F*(1, 121) = 6.45, *p* = 0.012, η_p_^2^ = 0.051, such that dishonest reporters (*M* = 1.47, SD = 0.53) used more first-person plural pronouns than truthful reporters(*M* = 1.25, SD = 0.45). No other effects were significant in the control condition. In the Putative Confession condition, there was a significant main effect of Age, *F*(3, 95) = 3.48, *p* = 0.019, η_p_^2^ = 0.099, which was subsumed by a significant 3-way interaction, *F*(3, 95) = 2.76, *p* = 0.046, η_p_^2^ = 0.080. Follow-up ANOVAs were conducted to further examine this interaction; however, when further split to examine significant effects of Age, Honesty, and Maltreatment, these ANOVAs revealed no significant differences.

### Predicting veracity

The final analysis involved using a binary logistic regression to predict dishonest and truthful reporters using the linguistic and syntactic variables on which they significantly differed. Specifically, first-person plural pronouns, cognitive mechanism terms, and syntactic complexity were entered as predictors with Honesty as the dependent variable (0 = truth-tellers, 1 = dishonest reporters). The overall model was significant in predicting truth-tellers and dishonest reporters, *χ*^2^ (3, N = 248) = 61.43, Nagelkerke *R*^2^ = 0.30, *p* < 0.001, with 74.2% of children being correctly classified. Interestingly, only syntactic complexity emerged as a significant predictor above and beyond the common contribution of all other variables, such that as syntactic complexity decreased children were 8 times more likely to be dishonest, *B* = −2.09, Wald = 37.29, *p* < 0.001, OR = 8.33. The use of cognitive mechanism terms, *B* = 0.06, Wald = 1.65, *p* = 0.199, OR = 1.06, and of first-personal plural pronouns, *B* = 0.109, Wald = 0.96, *p* = 0.328, OR = 1.12, did not uniquely predict group membership.

## Discussion

The current study examined linguistic and syntactic differences in maltreated and non-maltreated children’s truthful and dishonest coached reports of an interaction with an adult. Children’s dishonest reports included significantly more first-person plural pronouns and cognitive mechanism terms and were significantly less syntactically complex compared to truthful reports. Importantly, only syntactic complexity significantly differentiated truthful and dishonest reporters above and beyond the common contribution of all other variables in a logistic regression. The remaining linguistic cues examined did not differ between truthful and dishonest reporters, but some differences emerged based on age, maltreatment status, and honesty promotion.

### Linguistic cues to dishonesty

The overarching goal of the current research was to examine how linguistic cues differed between truthful and dishonest reporters. Several important findings emerged. First, it was predicted that lie-tellers would use more first-person pronouns than truth-tellers, as has been found in previous research examining children’s dishonest reports (Brunet et al., 2013; Williams et al., 2014; Talwar et al., 2018). Given that children were discussing an event in which they co-transgressed with an adult, both plural and singular first-person pronouns were examined separately. Interestingly, consistent with previous findings (Williams et al., 2014) dishonest reporters used more first-person plural pronouns than truthful reporters, but no differences were found for singular pronouns. The increased use of first-person plural pronouns may be particularly relevant when children are coached to dishonestly conceal a co-transgression. In the present study, children were coached to dishonestly report an event during which they played games and transgressed with a confederate. Thus, children likely referred to both themself and the confederate when providing their report due to the nature of the paradigm. Additionally, they may have preferred plural pronouns in case the transgression was discovered; including the confederate in their report ensured the interviewer would know that both individuals participated and thus the child could not be solely blamed for the transgression. Future studies in which a child is solely responsible for a transgression and no coaching occurred are necessary to more completely understand the role of first-person singular pronouns.

It was also predicted that, due to differences in perceptual experiences, dishonest reporters would use more cognitive mechanism terms and fewer affect terms than truth-tellers. This prediction was only supported for cognitive mechanisms: dishonest reporters used more cognitive mechanism terms than truthful reporters. Previous research on linguistic cues suggests that lie-telling relies on cognitive processes to fabricate events that were not experienced, rather than sensory or affective processes that would be used to recall true events (Vrij et al., 2004; Evans et al., 2012; Williams et al., 2014). These processes are thought to be reflected in the language used; while this was supported in the current study in children’s use of cognitive mechanism terms, we did not find differences in the use of affect terms. This finding aligns with previous research on the Reality Monitoring approach suggesting that these cognitive and affective processes are not uniquely able to differentiate between truth and lie-tellers (Gancedo et al., 2021). This may be due to the event being reported; both the truth-tellers and dishonest reporters experienced the same event during which they played a game; thus, both groups would rely on the sensory and affective processes used for true memory recall and would not differ between groups. The dishonest reporters, however, (1) omitted an aspect of the event (the transgression) and (2) provided the coached details. Omission would not require a change in words used as they simply did not mention the transgression. However, providing the coached details may have led to the increased cognitive mechanism terms (e.g., cause, know, and ought) as they had to provide details that had not been experienced. Given this pattern of findings, it is important to continue to examine instances of dishonesty in which a child is coached to conceal an aspect of an event and provide false details. For example, when children are interviewed about transgressions like sexual abuse, they may be coached by their abuser to conceal the abuse while still honestly reporting some information about what happened while they were together.

Contrary to predictions, we failed to find differences in the use of tentative and negation terms. In the only previous study to examine tentative terms with children, consistent with our findings, no significant differences were found between truth-and lie-tellers (Brunet et al., 2013), suggesting that tentative terms may not be a helpful cue in examining the veracity of children’s reports. Negation terms have been shown to be used more by adults in false reports (Ali and Levine, 2008; Hauch et al., 2015), but have not been examined in children’s reports. It was expected that perhaps children would use more negation terms to ensure the experimenter knew that they were not involved in the transgression (“I did *not* touch the button). This, however, was not the case; it appears that children may use language besides negation terms to accomplish this goal. For example, perhaps they blame others rather than emphasizing that they were not involved (Evans et al., 2021).

### Syntactic complexity and word count

The current study is the first to examine syntactic complexity as an indicator of dishonesty. Consistent with predictions, dishonest reporters used simpler sentence structure than truthful reporters. Given that lie-telling is a cognitively demanding task for children, they may devote cognitive resources to their report by monitoring what details they provide and ensuring they do not reveal the transgression. This may result in children using more simple statements, as these may be easier for them to monitor and ensure they conceal the relevant details. Future studies could test this explanation by examining whether the increased cognitive load results in simpler sentence structure by increasing children’s cognitive load when they report on an event.

It should be noted that there were also developmental findings; older children’s and non-maltreated children’s statements were more complex than younger and non-maltreated children’s statements, respectively. Given these developmental findings, complexity may be a less reliable indicator of dishonesty; understanding how complex a child’s report *should* be given their age would be important for examining whether their report is too simplistic to be truthful. Thus, future research should continue to examine syntactic complexity as an indicator of children’s dishonesty to understand how this may be useful in a practical context.

Unlike complexity, word count did not differ between truthful and dishonest reporters. Some studies have found that dishonest reports are shorter than truthful ones (Brunet et al., 2013), and some approaches, such as CBCA, use report length as an indicator of dishonesty (Vrij, 2005). However, word count differences have typically been found in studies where children fabricate the full event without being coached (Brunet et al., 2013). When children are coached to fabricate their full report, word count differences have not emerged (Evans et al., 2012; Saykaly et al., 2013; Williams et al., 2014; Talwar et al., 2018). In the present study, children (1) experienced the event and thus had the same amount of information as truthful reports and (2) were coached on details to provide and conceal. The coaching they received likely allowed them to provide a similar amount of information as the truthful reporters, leading their reports to be of similar length. This is an important finding given that when children are interviewed about events, it is unlikely that they will fabricate an entire event. Additionally, if they fabricate parts of an event and conceal some details, it is likely that they will have been coached by an adult to do so, particularly in cases of maltreatment. Previous research and the current study suggest that in these cases, word count is not a reliable indicator of dishonesty; when children receive some support to fabricate a cover story they will be able to provide the same amount of information as a child who tells the truth.

### Predicting dishonest vs. truthful reports

Given the differences found between truthful and dishonest reporters, we examined the extent to which the indicators that differed between the two groups could be used to predict group membership (cognitive mechanism terms, syntactic complexity, and exclusive terms). We found a higher rate of accuracy that is typically found in human lie detection research (~50%; Gongola et al., 2017). Interestingly, only syntactic complexity emerged as a significant predictor; as complexity decreased, children were 8 times more likely to be classified as dishonest reporters. This finding suggests that syntactic complexity may be a new, effective method for detecting deception in children. While the model predicted about 74% of children’s group membership accurately, it could be that finding other linguistic indicators of dishonesty in this type of paradigm would improve this model’s ability to predict deception. Future research should focus on a broader range of linguistic indicators to explore how to improve this model’s ability to predict truth and lie-tellers.

### Maltreatment

Interestingly, we did not find any differences in indicators of dishonesty between the maltreated and non-maltreated samples. The lack of differences is somewhat surprising given that maltreated children’s language development often differs significantly than non-maltreated children, both in terms of the scope of words learned and the complexity of their speech (Coster et al., 1989; Eigsti and Cicchetti, 2004; Sylvestre et al., 2016). Despite this, it is important to acknowledge that this finding is positive; maltreated children do not differ significantly in the types of words that are used when providing dishonest reports, and thus the indicators that have been found in previous research are likely also evident in maltreated children. However, it may be the case that we did not find differences because of coaching; coaching may have supported maltreated children in producing similar statements to that of non-maltreated children. Future research should examine whether this is the case by comparing maltreated children’s reports with and without coaching.

It is important to note that identifying linguistic or syntactic patterns to identify when children are being dishonest are also useful to identify when children are being honest. Identifying methods for differentiating truth and lie-tellers is useful for identifying instances of false allegations, honest or credible reports of abuse, as well as children who are lying to conceal abuse. Identifying children experiencing maltreatment, both by knowing when they are concealing and when they are honestly reporting, is vital for ensuring children are protected when necessary. These cues, specifically the use of first-person plural pronouns, cognitive mechanism terms, and syntactic complexity, may aid in identifying these cases.

### Limitations

There are several limitations of the current study to note. Children’s language proficiency was not assessed. Children with poorer language development (regardless of maltreatment) may have had less complex reports overall. Future studies should aim to account for children’s language proficiency. Similarly, the results likely do not generalize to other languages. The rules governing the syntactic structure of sentences varies across languages; thus, syntactic complexity may look different depending on language.

Another important limitation lies in the laboratory design (simulated transgression paradigm). These paradigms are useful in that the ground truth is known, so researchers can know with certainty which children are being truthful and which are being dishonest. However, these designs may lack external validity, particularly when being applied to reports of maltreatment, given the difference in the nature of the experience. Additionally, children may adjust their behavior in an experimental setting and not report on an event in the same manner they would during a forensic interview. Furthermore, the current study used an interview protocol based on the NICHD Structure Protocol, an interview which emphasizes the use of broad open-ended requests for recall. It is possible that the linguistic structure of children’s honest and dishonest reports may vary based on the interview protocol used. Thus, in the future, researchers should examine whether the current study’s findings replicate with other interview protocols.

### Conclusion

The present investigation found support for children’s use of first-person plural pronouns and cognitive mechanisms terms as an indicator of dishonesty. The current study also identified a novel indicator of dishonesty, syntactic structure, which was highly accurate in classifying truthful and dishonest reports. This finding suggests an additional cue to examine when detecting deception in children, although further research is needed to be able to use this to discover a threshold of complexity that might distinguish truth and lie-tellers. Furthermore, the current findings suggest that, for the cues examined, linguistic cues to dishonesty may not differ for maltreated and non-maltreated children, providing the first evidence that previous research using linguistic cues is useful for both populations.

## Data availability statement

The original contributions presented in the study are included in the article/supplementary materials, further inquiries can be directed to the corresponding author.

## Ethics statement

The studies involving human participants were reviewed and approved by University of Southern California institutional review board. Written informed consent to participate in this study was provided by the parent/legal guardian or Court/attorneys (maltreated children consent given by court).

## Author contributions

All authors listed have made a substantial, direct, and intellectual contribution to the work and approved it for publication.

## Funding

This work was supported by the National Institute of Child Health and Human Development (HD087685 and HD101617) and Social Sciences and Humanities Research Council of Canada.

## Conflict of interest

The authors declare that the research was conducted in the absence of any commercial or financial relationships that could be construed as a potential conflict of interest.

## Publisher’s note

All claims expressed in this article are solely those of the authors and do not necessarily represent those of their affiliated organizations, or those of the publisher, the editors and the reviewers. Any product that may be evaluated in this article, or claim that may be made by its manufacturer, is not guaranteed or endorsed by the publisher.
